# Attachment and Detachment of Living Microorganisms Using a Potential-Controlled Electrode

**DOI:** 10.1007/s10126-013-9495-2

**Published:** 2013-02-19

**Authors:** Sumihiro Koyama, Masa-aki Konishi, Yukari Ohta, Tetsuya Miwa, Yuji Hatada, Takashi Toyofuku, Tadashi Maruyama, Yuichi Nogi, Chiaki Kato, Taishi Tsubouchi

**Affiliations:** 1 Institute of Biogeosciences, Japan Agency for Marine-Earth Science and Technology, 2-15 Natsushima-cho, Yokosuka, Kanagawa 237-0061 Japan; 2 Marine Technology and Engineering Center, Japan Agency for Marine-Earth Science and Technology, 2-15 Natsushima-cho, Yokosuka, Kanagawa 237-0061 Japan

**Keywords:** Electrical modulation, Electrical attachment, Electrical detachment, Soil microorganisms, Deep-sea microorganisms, *Escherichia coli*

## Abstract

We developed an electrical modulation method for attachment and detachment of microorganisms. Living microorganisms suspended in non-nutritive media such as PBS(−) and artificial seawater were attracted by and selectively attached to indium tin oxide (ITO)/glass electrode regions to which a negative potential was applied. The microorganisms suspended in LB medium and glucose solution were not attracted to the ITO electrode. Dead microorganisms were not attracted to the ITO electrode. The living microorganisms were retrieved after detachment from the ITO electrode by application of a high-frequency triangular wave potential. When we applied this method to separate microorganisms from deep-sea sediment, bacteria belonging to 19 phyla and 23 classes were collected without undesirable high molecular weight contaminants such as humic acids. At the phylum and class level, respectively, 95 and 87 % of the phylotypes among electrically retrieved bacteria were common to the gene clones from the direct sediment DNA extraction. This technique is a novel useful method to prepare bacterial cells in a single population or a community for metagenomic analyses.

## Introduction

Microorganisms in soil and sediments are rich sources of novel therapeutic compounds such as antibiotics (Raaijmakers et al. 1997), anticancer agents (Shen et al. 2001), and immunosuppressants (Skoko et al. 2005), as well as a wide range of biotechnologically valuable products (Hatada et al. 2011; Ohta and Hatada 2006). However, the majority of microorganisms present in sediment and soil cannot be cultured under conventional laboratory conditions (Rajendhran and Gunasekaran 2008).

Metagenomics has been developed over the past decade to clarify a previously unknown diversity of microorganisms on the one hand; on the other hand, it has been driven by the increasing biotechnological demand for novel enzymes and biomolecules (Rajendhran and Gunasekaran 2008; Simon and Daniel 2011). Analysis of DNA directly extracted from sediment and soil samples has paved the way to studying natural microbial communities without the need for cultivation. However, high DNA yields and purity are difficult to achieve due to the co-extraction of humic substances that inhibit downstream applications, such as polymerase chain reaction (PCR), restriction enzyme digestion, and DNA ligation (Roh et al. 2006). In indirect DNA extraction methods, microorganisms are separated from the sediment and soil samples prior to cell lysis. Higher molecular weight and purer environmental DNA is obtained from indirect cell lysis compared with direct protocols (Courtois et al. 2001; Gabor et al. 2003; Roh et al. 2006). However, it was reported that the DNA obtained is usually derived from only about 25 to 35 % of the total number of microorganisms present in the soil and sediment samples (Rajendhran and Gunasekaran 2008). Various bacterial groups strongly attach to soil aggregates, which might bias the picture of the organization of the microbial community in the sample (Rajendhran and Gunasekaran 2008; Steffan et al. 1988).

Microbial single-cell isolation techniques have also been developed during the past decade for three major purposes: (1) to cultivate previously uncultured microbes, (2) to assess and monitor cell physiology and function, and (3) to screen for novel microbiological products such as enzymes and antibiotics (Brehm-Stecher and Johnson 2004; Broude 2011; Ishii et al. 2010). Microbial single-cell isolation techniques fall into five major categories (Ishii et al. 2010). The first is the dilution-to-extinction method, which is probably the simplest method to obtain single cells from heterogeneous populations and involves serial dilutions of a sample solution until only single cells remain (Button et al. 1993; Schut et al. 1993). The second is micromanipulation, which obtains single cells using a mechanical micromanipulator or an optical tweezer (Fröhlich and König 2000; Huber et al. 2000; Ishii et al. 2010). The trapped cells are subsequently used for cultivation or other analyses. The third is flow cytometry, which enables the rapid analysis of entire cell populations on the basis of single cell characteristics (Brehm-Stecher and Johnson 2004). Multiple characteristics including cell count, cell size or content, and responses to fluorescent probes diagnostic of cell function may be collected simultaneously in this method (Brehm-Stecher and Johnson 2004). The fourth is microfluidics, which isolates and incubates single cells of interest using a number of microflow channels (Fu et al. 1999; Hu et al. 2005). The fifth is compartmentalization of single cells, which are screened for potentially novel enzymes and for cultivation of yet-uncultured microbes (Bergquist et al. 2009; Bershtein and Tawfik 2008; Ishii et al. 2010; Link et al. 2007). However, it is extremely difficult to use these five techniques for direct assay of various living microorganisms in soil and/or sediment samples. Because the microorganisms adhere strongly to soil aggregates, it is difficult to separate them alive from the aggregates (Rajendhran and Gunasekaran 2008; Steffan et al. 1988).

In the present study, we demonstrated that a weak negative electric potential attracted living microorganisms to the electrode surface and that they were separated from sediment and soil particles. Furthermore, the electrically attached living microorganisms could be detached from the electrode by application of a high-frequency triangular wave potential. Using the electrical modulation technique, attachment and detachment of specifically positioned microorganisms can be modulated iteratively at the same positions on the electrode surface.

## Materials and Methods

### Soil and Deep-Sea Sediment Samples

The surface layer of the deep-sea sediment sample was collected by the unmanned submersible *Hyperdolphin* (dive 1237; January 20, 2011; 1,176 m; 35°00.13′ N, 139°13.51′ E) from the seep area of Sagami Bay, Japan (Koyama and Aizawa 2000; Koyama et al. 2005). The garden soil sample was collected from the grapery of the Japan Agency for Marine-Earth Science and Technology.

### Bacterial Strains and Media

*Bacillus halodurans*, *Shewanella violacea*, *Escherichia coli*, *Bacillus subtilis*, *Shewanella oneidensis*, and *Kocuria rosea* were used as test microorganisms. *B*. *halodurans* C-125, formerly known as *Bacillus* sp. strain C-125 (Takami and Horikoshi 1999), was thawed and recovered using Horikoshi I medium (Horikoshi and Akiba 1982) and then grown aerobically at 37 °C in Horikoshi I medium. *S*. *violacea* DSS12^T^, isolated from the Ryukyu trench, was thawed and regenerated using an autoclaved and 0.22-μm filtered Marine broth 2216 medium (Difco Laboratories, Detroit, MI, USA) (Kato et al. 1995). The *S*. *violacea* cells were grown aerobically at 8 °C in the filtered Marine broth 2216 medium. *E*. *coli* (JCM1649^T^) and *B*. *subtilis* (JCM1465^T^) were grown aerobically at 37 °C in Luria–Bertani (LB) medium (Difco Laboratories). *S*. *oneidensis* (ATCC770550^T^) was grown aerobically at 30 °C in LB without NaCl (LB△N) medium containing tryptone 10.0 g l^−1^ and yeast extract 5.0 g l^−1^. *K*. *rosea* (NBRC 3768^T^) was grown aerobically at 30 °C in LBNG medium containing (g l^−1^): glucose, 30.0; tryptone, 10.0; yeast extract, 5.0; and NaCl, 30.0. The glucose was sterilized separately and then added to the medium.

### Electrode Preparation

**Fig. 1:**
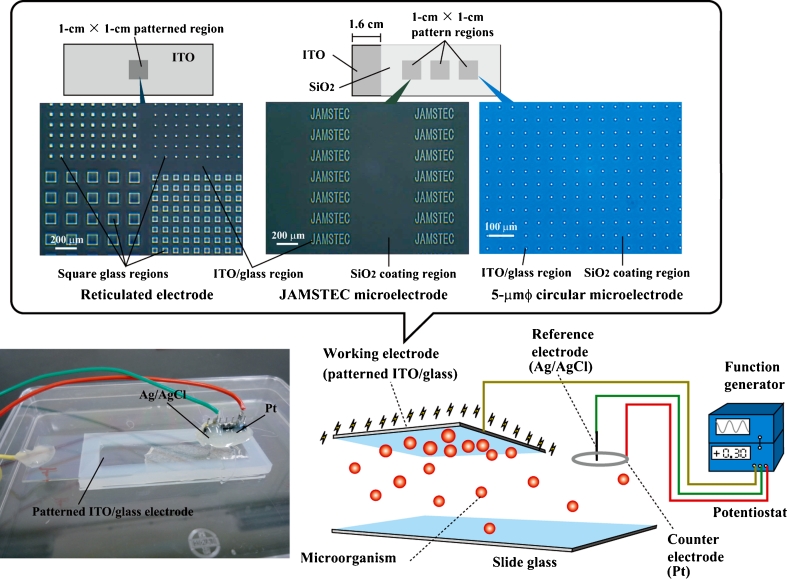
Schematic illustration of a patterned indium tin oxide (*ITO*)/glass electrode culture system. The electrode potential is controlled with an Ag/AgCl reference electrode through a potentiostat and a function generator

Patterned working electrodes were constructed by vacuum evaporation of indium tin oxide (ITO; In_2_O_3_; 10 Ω/cm^2^) and an insulator of silicon dioxide (SiO_2_) onto 76 × 26-mm^2^ silica glass plates (1 mm in thickness) (Geomatec Co., Ltd., Yokohama, Japan). The reticulated ITO electrode with arrayed square glass regions (Fig. 1) was described elsewhere (Koyama 2011). The microelectrode was formed by the plane ITO electrode fabricated with a coating of SiO_2_ (Fig. 1). The 76 × 26-mm^2^- and 5-mm-thick silicon rubber plate with a hollow interior measuring 66 × 16 mm^2^ was glued to the 76 × 26-mm^2^ slide glass by silicon bonding (Fig. 1). The patterned ITO/glass electrode was attached to the top of the silicon rubber box (Fig. 1). The fabricated silicon rubber box was housed in a sterile square plastic dish. A 12-mm-diameter section of both the Pt ring counter electrode and Ag/AgCl reference electrode was placed on the plastic lid of the square plastic dish (Fig. 1).

For viability analyses of the electrically detached microorganisms, we used a previously described three-electrode chamber system (Koyama 2011). The plastic chamber section of a Lab-tek chamber slide system (Cat. 177410, NalgeNunc International, Naperville, IL, USA) was glued to the patterned ITO/glass electrode with silicon bonding. The Pt counter electrode and Ag/AgCl reference electrode were placed on the plastic lid of the chamber slide system.

Large electrode chamber devices were constructed for phylogenetic analyses of the microorganisms in sediment. A 110 × 85-mm^2^- and 5-mm-thick silicon rubber plate with a hollow interior measuring 90 × 65 mm^2^ was glued to a 125 × 85-mm^2^ plane ITO/glass electrode with silicon bonding. The large plane ITO/glass electrode was placed on the bottom of the large electrode chamber device and housed in a sterile square plastic dish. Thirty-millimeter-diameter sections of the Pt ring counter electrode and Ag/AgCl reference electrode were placed on the plastic lid of the square plastic dish.

Both the patterned and the large ITO/glass electrodes were sonicated in ultrapure water for 5 min and immersed in 1 M NaOH for 5 min to remove any unwanted deposits. After the procedure, the electrodes were washed with ultrapure water and dried. Then, the three electrode chambers were irradiated with UV light for 5 min for sterilization.

### Potential Application

Constant and triangular potentials were applied to the working ITO/glass electrode using the Ag/AgCl reference and the Pt counter electrode (Fig. 1). The constant potential application was done using a potentiostat (PS-14, Toho Technical Research, Tokyo, Japan). For 9-MHz triangular wave potential application (Koyama 2011), a coaxial cable was rolled up three times and made to a 10-cm-diameter coil. The coaxial cable coil connected between a function generator (AD-8624A, A&D Company, Tokyo, Japan) and the potentiostat (Toho Technical Research). The output potentials were checked by a digital oscilloscope (MT-770, Xiamen Lilliput technology Co., Ltd., Fujian, China). The function generator generated 0 ~ ±1.0 V 3-MHz rectangular wave signals linearly changed to 0 ~ ±10-mV vs. Ag/AgCl 9-MHz triangular wave potentials by impedance of both the coiled coaxial cable and the potentiostat. The function generator generated ±4.0 V 3-MHz rectangular wave signal which was necessary to obtain ±20-mV vs. Ag/AgCl 9-MHz triangular wave potential. The 9-MHz triangular wave potential was used for microbial detachment experiments.

For attachment of the microorganisms to the patterned ITO/glass electrode regions (Fig. 1), a constant potential was applied to the electrode for 24 h. The cultured microorganisms and soil or sediment samples were suspended in either Dulbecco’s PBS(−) (Wako, Osaka, Japan), MOPS buffer (10 g/L; pH 7.0, Dojindo, Kumamoto, Japan), tricine buffer (10 g/L; pH 7.0, Dojindo), 280 mM glucose solution (10 g/L), LB medium, or artificial seawater (30 g of NaCl, 0.7 g of KCl, 5.3 g of MgSO_4_ · 7H_2_O, 10.8 g of MgCl_2_ · 6H_2_O, 1 g of CaCl_2_ · 2H_2_O per liter). Each of the test microorganisms was allowed to grow overnight, then centrifuged for 2 min at 2,150 × *g* and 4 °C, and replaced in the selected solution at each cultivation temperature. In the electrical attachment experiments with dead *E*. *coli*, the pellet was resuspended in 70 % EtOH with vortexing and incubated for 1 h at 60 °C. After 70 % EtOH fixation, the *E*. *coli* cells were centrifuged for 2 min at 2,150 × *g* and 4 °C and then replaced in PBS(−). The microorganism suspensions were diluted to a concentration of 1 × 10^6^ cells/5 ml and poured into the three-electrode chamber system (Fig. 1). In the experiments with sediment and soil samples, 0.1 g/ml of the garden soil in the selected solution at room temperature (RT) or 0.1 g/ml of the deep-sea sediment in artificial seawater at 4 °C was suspended with 5-min vortexing. The suspended soil or sediment samples were diluted to a concentration of 50 μg/5 ml in the selected solution and then poured into the chamber system. To detach the microorganisms from the electrode surface, ±10-mV vs. Ag/AgCl 9-MHz triangular wave potential was applied to the electrode in either PBS(−) at RT or artificial seawater at 4 °C for 60 min. Under anaerobic experimental conditions, the electrical attachment of microorganisms was performed using an anaerobic cultivation system (Anaero pack A-07 with anaerobic jar, Mitsubishi Gas Chemical Company, Inc., Tokyo, Japan). A hole was drilled through the lid of the anaerobic jar to allow three conductive wires to penetrate, and the hole was then blocked with epoxy resin bonding. The three-electrode chamber system connected to the conductive wires through the lid was housed in the anaerobic cultivation system.

For phylogenetic analyses of microorganisms in soil or sediment samples, microorganisms were collected from the sample using the large electrode chamber device. The microorganisms collected were purified twice using an electrical retrieval method. One gram per milliliter of the soil or the sediment in either PBS(−) at RT or artificial seawater at 4 °C was treated with 5-min vortexing. To attach the microorganisms in the soil or sediment sample to the large ITO/glass electrode, 12.5 ml of the suspended sample in either PBS(−) at RT or artificial seawater at 4 °C was poured into the large chamber. In the deep-sea sediment sample, a −0.3 V vs. Ag/AgCl constant potential was applied to the large electrode for 2 h at 4 °C in artificial seawater. In the garden soil sample, −0.4 V vs. Ag/AgCl constant potential was applied to the large electrode for 2 h at RT in PBS(−). After 2 h of application, the electrode was washed three times with either artificial seawater at 4 °C or PBS(−) at RT, and the microorganisms attached to the electrode were detached by applying ±10-mV vs. Ag/AgCl 9-MHz triangular wave potential for 30 min in 12.5 ml of either fresh artificial seawater at 4 °C or fresh PBS(−) at RT. After the triangular wave potential application, the detached microorganisms were collected with a cell scraper and transferred to a new large electrode chamber device, and −0.3 V or −0.4 V vs. Ag/AgCl constant potential was applied for a further 2 h at 4 °C in seawater or at RT in PBS(−). After application, the electrode was washed three times, and the microorganisms on the electrode were detached by application of ±10-mV vs. Ag/AgCl 9-MHz triangular wave potential for a further 30 min in 12.5 ml of either fresh artificial seawater at 4 °C or fresh PBS(−) at RT. After these procedures, the electrically collected microorganisms were used in phylogenic analyses.

We observed a weak electrical current of −0.19 to −0.27 μA/cm^2^ when a negative constant potential between −0.3- and −0.4 V vs. Ag/AgCl was applied to the samples except when using 0.1 g/ml of sediment or soil suspension. We measured −0.79 to −0.88 μA/cm^2^ of the weak current using 0.1 g/ml of the sediment or soil suspension.

### Optical Microscopic Observation of Dehydrogenase-Active Microorganisms

To analyze the respiratory activity of the microorganisms attached to the electrode, we used a Bacstain CTC rapid staining kit for microscopy (Dojindo, Kumamoto, Japan). The respiratory activity staining solution was comprised of 20 μl of 50 mM CTC solution and 5 μl of enhancing reagent B added to 1 ml of either PBS(−) or artificial seawater. After vortexing the staining solution, the microorganisms on the electrode were incubated with the staining solution at cultivation temperature for 30 min and observed using the confocal laser scanning microscope system (FV500, Olympus, Tokyo, Japan).

### Analyses of Detachment, Survival, and Viable Bacteria Collection Rates

For measurement of detachment rates, cell numbers were counted at randomly selected 50 × 50-μm^2^ regions on the ITO electrode before and after triangular wave potential application. The detachment rate was calculated from the cell number after the triangular wave potential application divided by the cell number before the electrical application.

For measurement of survival rates, more than 300 of total microorganisms on the electrodes were distinguished using a live/dead backlight bacterial viability kit for microscopy and quantitative assays (L7012, Molecular Probes, Eugene, OR, USA) using the confocal laser scanning microscope system (FV500, Olympus) according to the manufacturer’s recommendations. The survival rates were calculated from the sum of three independent experiments.

In cell counts of microorganisms in soil, the suspended soil samples diluted to a concentration of 50 μg/5 ml in the selected solution were treated with the live/dead backlight bacterial viability kit for microscopy and quantitative assays. Then, the microorganisms in soil were counted with a hemacytometer under confocal laser scanning microscopic observation before and after high-frequency triangular wave potential application.

In the viable bacteria collection rate analyses of *B*. *subtilis* and *E*. *coli*, we collected the supernatants after the −0.4 V vs. Ag/AgCl potential and after the triangular wave potential applications. Colonies were formed on LB agar plates at 37 °C. The viable bacteria collection rate was calculated from the colony number after the triangular wave potential application divided by the total colony number. Data were obtained from the sum of two or three independent experiments.

### Atomic Force Microscopic Observation

*E*. *coli* attached to the microelectrode (Fig. 1) was fixed with 2.5 % glutaraldehyde (Sigma, St. Louis, MO, USA) in PBS(−) for 1 h at RT, rinsed with milliQ water (Millipore, Billerica, MA, USA), dried in a desiccator, and observed with an atomic force microscope (MFP-3D-Bio, Asylum Research, Santa Barbara, CA, USA) using the acoustic AC mode with a microcantilever (OMCL-AC240TS-C2, Olympus). All images were obtained with 256 scan points, 256 scan lines, and a scan rate of 0.36 Hz under atmospheric pressure at RT. Images were analyzed using MFP3D software (Asylum Research) and argyle light software (Asylum Research).

### Scanning Electron Microscopic Observation

Microorganisms attached to the patterned ITO/glass electrode were prefixed with 2.5 % glutaraldehyde in PBS(−) for 1 h at each cultivation temperature. For observations of *S*. *violacea*, conductive staining procedures were performed with artificial seawater instead of PBS(−). After washing with PBS(−) three times for 10 min each, the microorganisms were postfixed with 2 % osmium tetraoxide in PBS(−) for 2 h at 4 °C. After washing with distilled water at 4 °C six times for 10 min each, conductive staining was performed by incubating with 0.2 % aqueous tannic acid (pH 6.8) at 4 °C for 30 min. The microorganisms were washed with distilled water at 4 °C six times for 10 min each and then treated with 1 % aqueous tannic acid at 4 °C for 30 min. After washing with distilled water at 4 °C six times for 10 min each, the microorganisms were dehydrated in a graded ethanol series and critical point-dried (JCPD-5 critical point drier, Japan Electron Optics Laboratories Ltd., Tokyo, Japan). The microorganisms on the electrode were coated with osmium using an osmium plasma coater (POC-3, Meiwa Shoji Co., Osaka, Japan) and observed with a field emission scanning electron microscope (JSM-6700 F, Japan Electron Optics Laboratories Ltd.) at an acceleration voltage of 5 kV.

### DNA Extraction, Small Subunit rRNA Gene PCR Amplification, and Phylogenetic Analyses

Environmental genomic DNA from the deep-sea sediments was extracted using Isoil Large for Beads ver. 2 (Nippon Gene Corp., Toyama, Japan) with slight modifications (100 μg/ml lysozyme was added to the lysis solution and incubated for 60 min). DNA marker used an all purpose Hi–Lo DNA marker (Bionexus, Inc., Oakland, CA, USA). Genomic DNA of the electrically isolated microorganisms in the deep-sea sediment was extracted using the CTAB method. The nearly whole-length bacterial small subunit SSU rRNA gene fragments were amplified from the environmental genomic DNA with general primers 27 F (5′-AGAGTTTGATCMTGGCTCAG-3′) and 1492R (5′-GGCTACCTTGTTACGACTT-3′) (Lane et al. 1985). Each 25-μl aliquot of the reaction mixture contained 2.5 μl of 10 × PCR buffer (Takara Bio Inc., Otsu, Japan), 4 μl of dNTP mix (2.5 nM each), 1 μl of 27 F primer (10 μM), 1 μl of 1492R primer (10 μM), 2.5 μl of MgCl_2_ solution (25 mM), 0.25 μl of *LA Taq* polymerase, 1 μl of template DNA, and RNase/DNase-free water to a final volume of 25 μl. The following PCR program was used: 96 °C for 5 min, followed by 25 cycles of 96 °C for 20 s; 55 °C for 15 s; and 72 °C for 90 s, followed by 72 °C for 7 min. The products were checked by electrophoresis, purified using QIAquickGel Extraction Kit (Qiagen, Valencia, CA, USA), and subsequently cloned into a pT7Blue-2 (Merck Chemicals, Darmstadt, Germany) vector using Competent High DH5α (Toyobo, Osaka, Japan) as the host. Approximately 130 clones of each library containing the fragments of expected length were selected randomly and sequenced using a BigDye Terminator v3.1 Cycle Sequencing Kit (Life Technologies Corp., Carlsbad, CA, USA) in a 3730*xl* DNA analyzer following the manufacturer’s recommendations. Sequences representing different phylotypes were deposited in the GenBank database with accession numbers AB687722–AB687989.

Sequences were checked with both Mallard software (Ashelford et al. 2006) and the CHECK_CHIMERA tools from the Ribosomal Database Project II (http://rdp8.cme.msu.edu/cgis/chimera.cgi?su = SSU), and chimeric sequences were excluded from further analyses. The closest relatives of the remaining sequences were obtained from the SILVA database (http://www.arb-silva.de/) using the BLAST program.

### Statistical Analysis

Statistical analysis was performed using Student’s *t*-test. The calculations were performed using Microsoft Excel.

## Results

### Electrical Attachment of *Escherichia coli* to a Patterned ITO Electrode

**Fig. 2:**
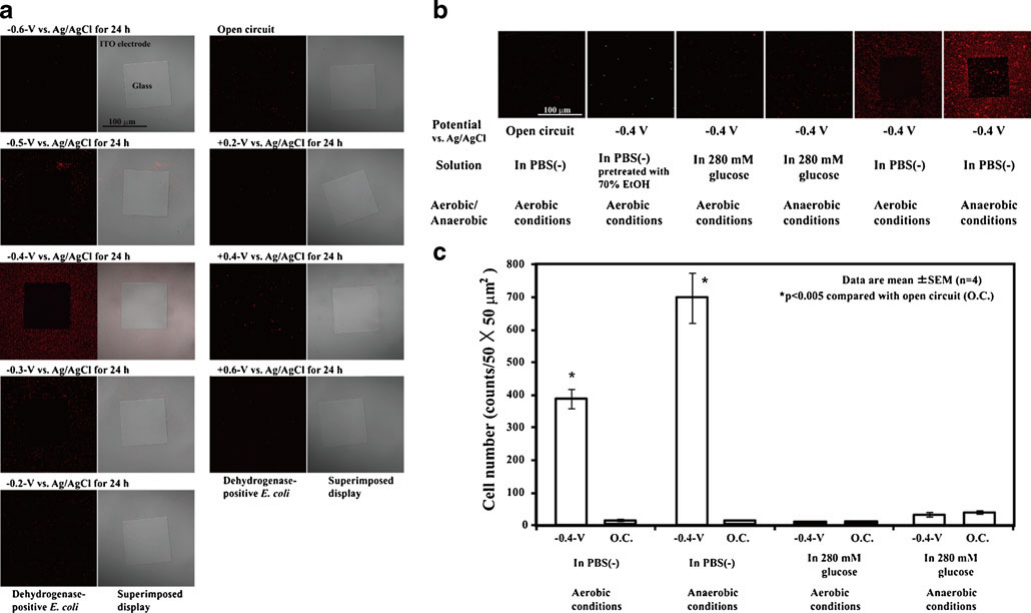
Attachment of *E*. *coli* cells to the constant potential applied electrode. **a** Distribution patterns of *E*. *coli* cells on the constant potential applied patterned ITO electrodes in PBS(−) for 24 h at RT. *E*. *coli* cells were stained with a Bacstain CTC rapid staining kit. **b** Distribution pattern and **c** cell density of *E*. *coli* on the patterned ITO electrode surface. *E*. *coli* cells pretreated with 70 % EtOH were stained with SYTO9. A −0.4 V vs. Ag/AgCl electrode was applied for 24 h in PBS(−) or 280 mM glucose under aerobic or anaerobic conditions at RT

To examine whether the microorganisms were attracted by and attached to a reticulated ITO/glass electrode region with an applied potential (Fig. 1), the gram-negative bacterium *E*. *coli* was used as a test microorganism. Cultured *E*. *coli* were washed with PBS(−) and diluted to a concentration of 1 × 10^6^ cells/5 ml in PBS(−) at RT and then poured into the three-electrode chambers on a glass slide (Fig. 1). To analyze the respiratory activity of the microorganisms attached to the electrode, we used a Bacstain CTC rapid staining kit for microscopy. Cyano-ditolyl-tetrazolium chloride (CTC), a monotetrazolium redox dye that produces red fluorescent formazan when it is chemically or biologically reduced as in the presence of dehydrogenase activity, was used as an indicator of respiration (Frederiks et al. 2006; Hiraishi and Yoshida 2004). Confocal laser scanning microscopy showed that the fluorescent formazan was exclusively localized at the surface of individual cells and not at intracellular sites (Frederiks et al. 2006). Figure 2a shows the distribution pattern of *E*. *coli* on the patterned ITO electrode after 24 h of constant potential applications. A constant potential between +0.6 and −0.6 V vs. Ag/AgCl was applied to the patterned ITO electrode in PBS(−) for 24 h at RT. After the potential applications, living *E*. *coli* on the electrode was stained with CTC to determine respiratory activity, and red fluorescent formazan production was observed using the confocal laser scanning microscope. Living *E*. *coli* was attracted by and selectively attached to the reticulated ITO electrode surface to which a negative potential between −0.3 and −0.5 V vs. Ag/AgCl was applied (Fig. 2a). A −0.4 V vs. Ag/AgCl constant potential application induced the maximum attachment of living *E*. *coli* to the reticulated ITO electrode region (Fig. 2a). Although most microorganisms including *E*. *coli* strains have a negative zeta potential under neutral pH (Bayer and Sloyer 1990; Ebersole and McCormick 1993), few or no *E*. *coli* cells selectively attached to the ITO electrode region to which the positive potential was applied (Fig. 2a). In addition, we investigated whether dead *E*. *coli* cells selectively attached to the ITO electrode region to which a negative potential was applied. The cultured *E*. *coli* cells were treated with 70 % EtOH for 1 h at 60 °C and replaced in PBS(−) at RT. After fixation of the *E*. *coli* cells, −0.4 V vs. Ag/AgCl constant potential was applied to the patterned ITO electrode for 24 h at RT (Fig. 2b). Few or no dead *E*. *coli* cells were observed on the ITO electrode region to which the −0.4 V vs. Ag/AgCl potential was applied (Fig. 2b). To clarify the interaction between living *E*. *coli* and the negative applied potential electrode, we examined whether the *E*. *coli* cells in glucose or PBS(−) solutions and under aerobic or anaerobic conditions were attracted by and attached to the negative-potential electrode (Fig. 2b, c). *E*. *coli* cells suspended in either 280 mM glucose solution or PBS(−) were poured into the three-electrode chamber system. A −0.4 V vs. Ag/AgCl potential was applied to the *E*. *coli* cells under either aerobic or anaerobic conditions for 24 h at RT. Figure 2b, c shows the density of the *E*. *coli* cells attached to the electrode surface. In PBS(−) conditions, −0.4 V vs. Ag/AgCl potential application enhanced the *E*. *coli* cell density on the reticulated ITO electrode region compared with the open circuit (Fig. 2b, c). The attached *E*. *coli* cell density was increased by 46- and 68-fold compared with the open circuit under aerobic and anaerobic conditions, respectively (Fig. 2b, c). In contrast to the enhanced *E*. *coli* cell density in PBS(−), no statistically significant differences between the −0.4 V vs. Ag/AgCl potential application and the open circuit were observed in 280 mM glucose under aerobic or anaerobic conditions (Fig. 2b, c). Figure 2 indicates that the principle of electrophoresis does not explain the phenomena of the negative potential-induced selective attachment of living *E*. *coli* to the reticulated ITO electrode region.

**Fig. 3:**
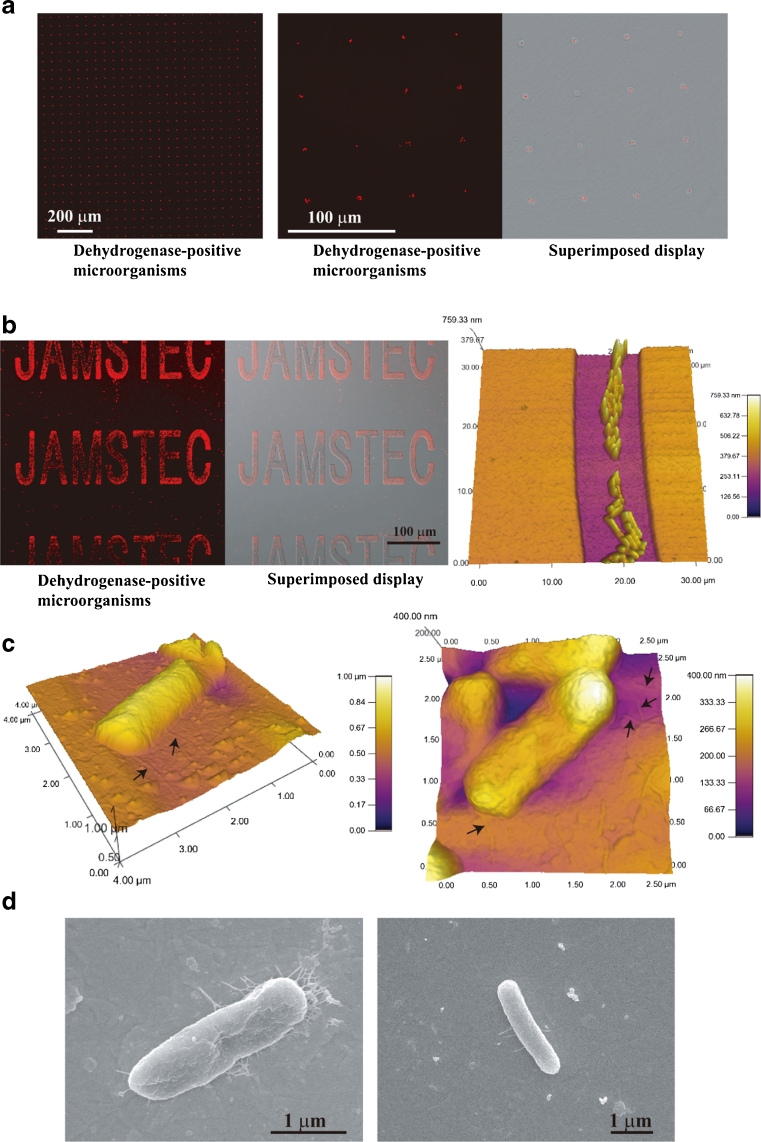
Fibrous elongated materials of *E*. *coli* cells attached to patterned ITO electrodes. **a** *E*. *coli* cells attached to 5-μm-diameter circular microelectrodes to which −0.4 V vs. Ag/AgCl potential was applied in PBS(−) for 15 h at RT. **b** *E*. *coli* cells attached to the JAMSTEC microelectrode to which −0.4 V vs. Ag/AgCl potential was applied in PBS(−) for 24 h at RT. Magnified AFM image is the middle region of “C.” **c** AFM images of *E*. *coli* cells attached to the JAMSTEC microelectrode. *Arrows* indicate elongated fibrous materials. **d** SEM images of *E*. *coli* cells attached to the reticulated ITO electrode

To perform accurate cell arrangement on the electrode surface, we examined whether *E*. *coli* cells recognized small regions of the negative applied potential microelectrode (Figs. 1 and 3a, b). Figure 3a, b shows living *E*. *coli* cells attached to small regions of the patterned ITO electrode to which −0.4 V vs. Ag/AgCl potential was applied at RT in PBS(−). We confirmed that a small number of *E*. *coli* cells selectively attached to the negative-potential 5-μm-diameter circular microelectrode array even if the electrode surface area was small compared with the reticulated electrode (Figs. 1 and 3a). Few or no *E*. *coli* cells were attached to the SiO_2_ coating region of the microelectrode array (Figs. 1 and 3a). The results in Fig. 3a clearly show that electrical modulation of the spatial configuration of *E*. *coli* cells was successful using the patterned ITO electrode culture system. To investigate how *E*. *coli* cells attached to the negative applied potential ITO electrode region, we observed the cells on the patterned electrode using a confocal laser scanning microscope, an atomic force microscope (AFM), and a scanning electron microscope (SEM) (Figs. 1 and 3). Figure 3b, c shows optical microscopic and AFM images of the *E*. *coli* cells attached to the electrode regions. The *E*. *coli* cells that attached to the electrode surface (Fig. 3c) had an elongated, short fibrous shape. We further examined the *E*. *coli* cells attached to the electrode surface using SEM (Fig. 3d). Figure 3d shows SEM images of *E*. *coli* cells on the negative applied potential electrode surface. *E*. *coli* cells appeared to adhere to the negative potential electrode surface with some elongated, short fibrous materials (Fig. 3c, d).

### Electrical Attachment of *B*. *subtilis*, *B*. *halodurans*, *K*. *rosea*, *S*. *violacea*, *S*. *oneidensis*, and Soil Microorganisms to the Patterned ITO Electrode

**Fig. 4:**
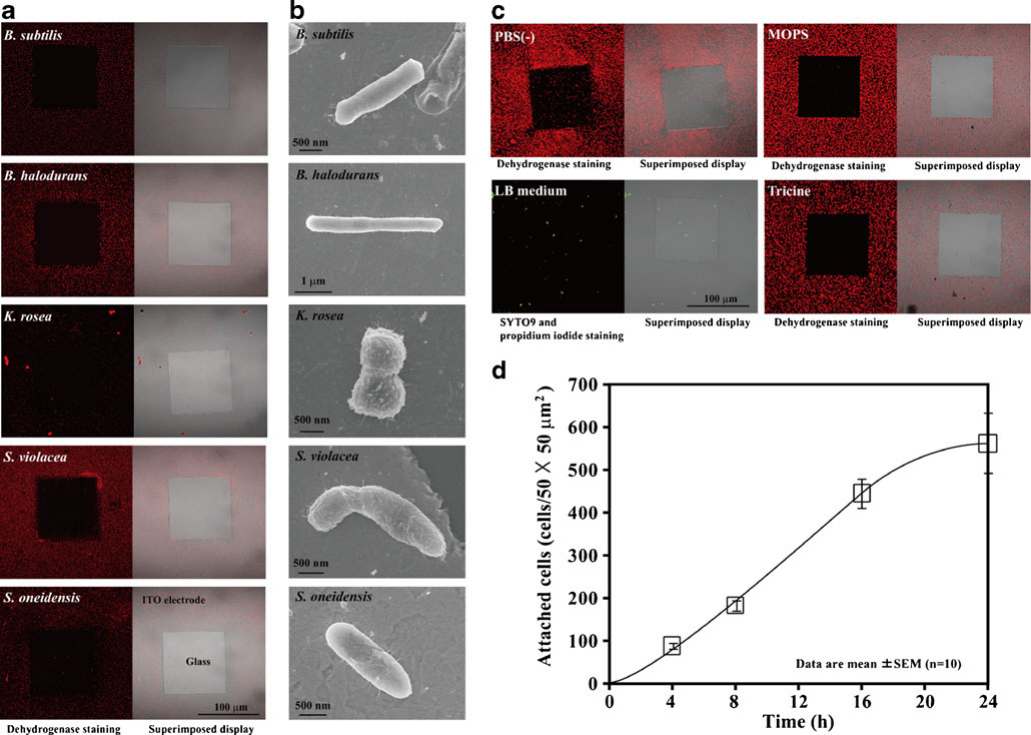
A negative potential attracted cultured and soil microorganisms to the patterned ITO electrode. **a** *B*. *halodurans*, *B*. *subtilis*, *S*. *oneidensis*, and *K*. *rosea* attached to the patterned ITO electrode to which −0.4 V vs. Ag/AgCl potential was applied for 24 h in PBS(−) at RT. *S*. *violacea* attached to the patterned ITO electrode to which −0.3 V vs. Ag/AgCl potential was applied for 24 h in artificial seawater at 8 °C. **b** SEM images of five different microorganisms attached to the patterned ITO electrode. **c** Distribution pattern of garden soil microorganisms on the patterned ITO electrode after 24 h of potential application in four different solutions. A −0.4 V vs. Ag/AgCl constant potential was applied at RT. **d** Time course of soil microorganism attachment to the electrode with −0.4 V applied potential in PBS(−) at RT

**Fig. 5:**
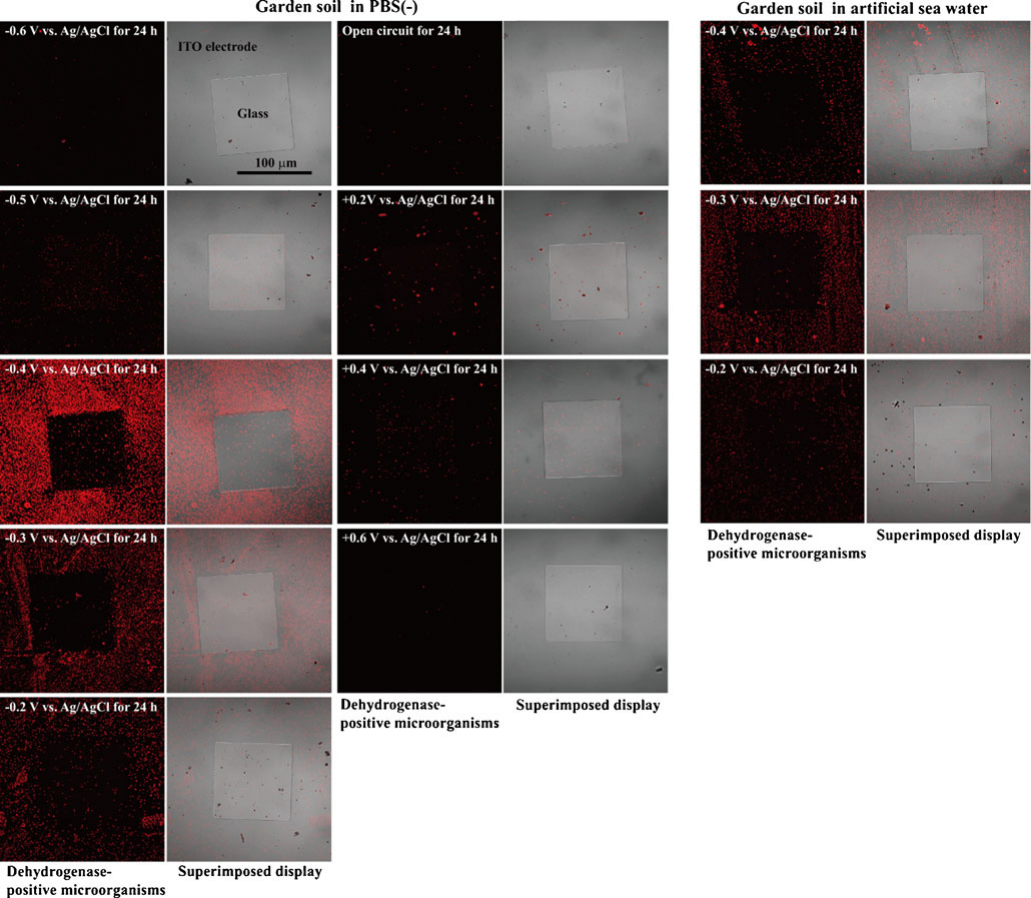
Distribution patterns of soil microorganisms attached to the patterned ITO electrode after 24 h of constant potential application at RT

We investigated whether a negative applied potential induced not only *E*. *coli* but also other microorganisms to attach to the reticulated ITO electrode. As test cells, we used one typical gram-positive bacterium, *B*. *subtilis*; two environmental bacteria, *B*. *halodurans* and *S*. *oneidensis*; one actinomycete, *K*. *rosea*; and one deep-sea bacterium, *S*. *violacea*. Figure 4a shows the distribution pattern of *B*. *subtilis*, *B*. *halodurans*, *K*. *rosea*, *S*. *violacea*, and *S*. *oneidensis* on the patterned ITO electrode after 24 h of constant potential application. They were attracted by and selectively attached to the reticulated ITO electrode surface to which a negative potential was applied (Fig. 4a). Except for *S*. *violacea*, −0.4 V vs. Ag/AgCl potential induced the maximum attachment of these organisms to the reticulated ITO electrode region (Fig. 4a). A −0.4 V vs. Ag/AgCl or lower applied potential in artificial seawater induced an adsorption wave of positive ions to the electrode surface, and the maximum attachment of *S*. *violacea* occurred a −0.3 V vs. Ag/AgCl applied potential (Figs. 4a and 5). Figure 4b shows SEM images of *B*. *subtilis*, *B*. *halodurans*, *K*. *rosea*, *S*. *violacea*, and *S*. *oneidensis* on the reticulated ITO electrode region. All five microorganisms also produced short fibrous materials that were very similar in both form and size to those of *E*. *coli* and attached to the negative applied potential electrode region (Figs. 3d and 4b).

Next, we examined whether microorganisms in soil were attracted by the negative applied potential electrode in various types of solution (Figs. 4c and 5). Garden soil samples were suspended in either PBS(−), MOPS buffer, tricine buffer, or LB medium and placed in the three-electrode chamber system. A −0.4 V vs. Ag/AgCl potential was then applied to the soil samples in the various solutions for 24 h at RT. Figure 4c shows the distribution pattern of the soil microorganisms on the negative applied potential electrode surface. The soil microorganisms in each of the non-nutritive buffers such as PBS(−), MOPS buffer, and tricine buffer selectively attached to the reticulated ITO electrode region to which the −0.4 V vs. Ag/AgCl potential was applied (Fig. 4c). The characteristic features of constant potential application that induced soil microorganism attachment were nearly identical to those that induced *E*. *coli* attachment (Figs. 2a and 5). The soil microorganisms in PBS(−) were attracted by and selectively attached to the reticulated ITO electrode surface to which a negative potential of between −0.2 and −0.4 V vs. Ag/AgCl was applied (Fig. 5). The maximum attachment of soil microorganisms was observed at −0.4 V vs. Ag/AgCl applied potential (Fig. 5). In artificial seawater, the maximum attachment of soil microorganisms was shifted to −0.3 V vs. Ag/AgCl because the adsorption wave of positive ions occurred at −0.4 V vs. Ag/AgCl on the electrode surface (Fig. 5). Meanwhile, few soil microorganisms in LB medium attached to the ITO electrode region to which −0.4 V vs. Ag/AgCl potential was applied (Fig. 4c). When the microorganisms in LB medium were examined in the cell viability test, it was confirmed that −0.4 V vs. Ag/AgCl applied potential was nearly non-cytotoxic, and 92 % (118 of 128 cells) of the soil bacteria remained alive on the electrode surface 24 h after the potential application (Fig. 4c). Figure 4d shows the time course of garden soil microorganism attachment in PBS(−) to the reticulated ITO electrode region. The −0.4 V vs. Ag/AgCl applied potential induced microorganism attachment that increased in a linear fashion until 16 h, after which the cell attachment rate became sluggish (Fig. 4d). The results in Figs. 2, 4, and 5 indicate that the microorganisms can attach to the electrode with a negative applied potential when the cells are suspended in non-nutritive media such as PBS(−), MOPS buffer, tricine buffer, and artificial seawater.

### Electrical Detachment of Microorganisms from the ITO Electrode

**Fig. 6:**
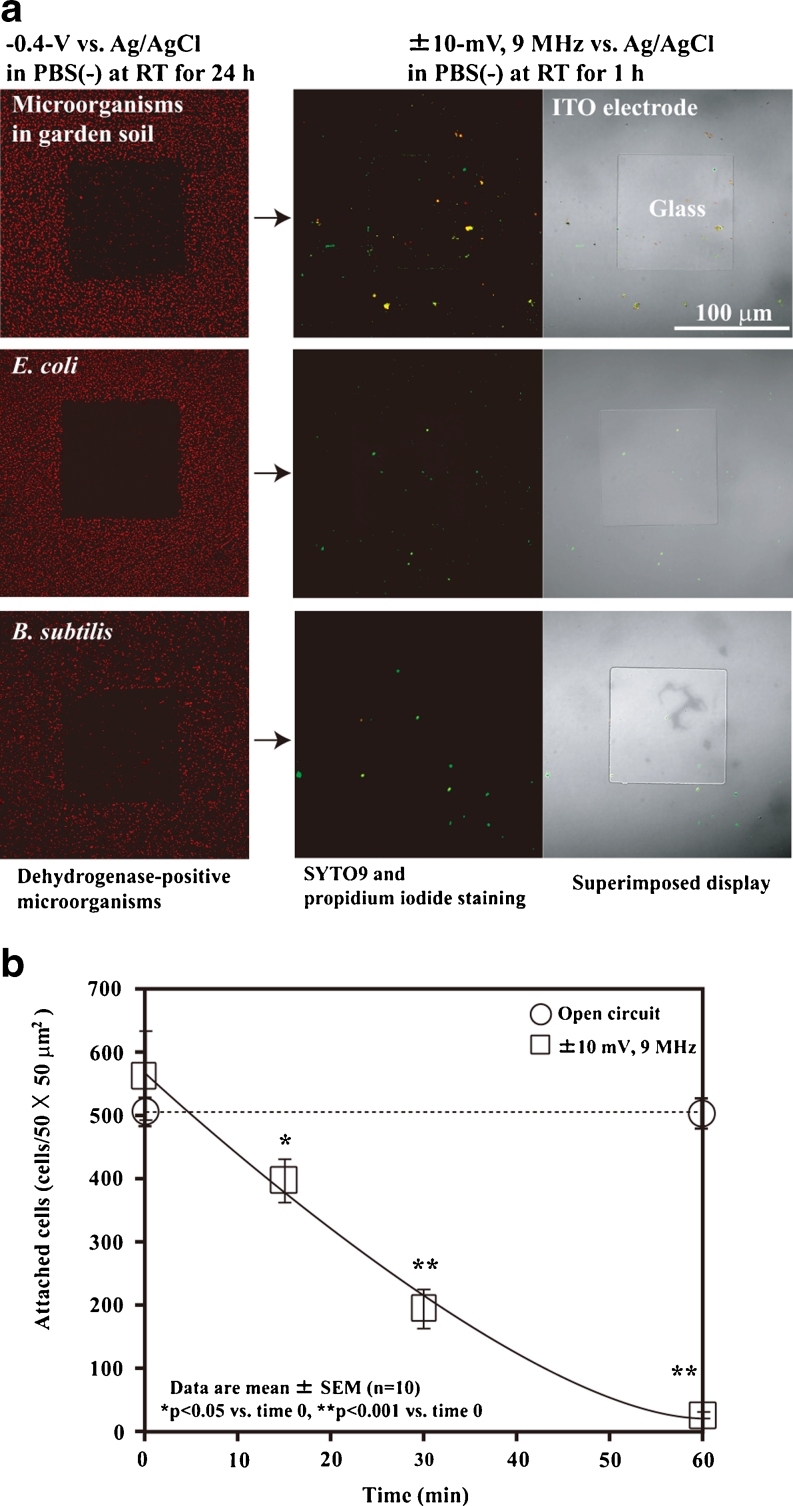
Electrical detachment of microorganisms. **a** Photographs of electrically detached soil microorganisms, *E*. *coli*, and *B*. *subtilis*. **b** Time course of soil microorganism detachment from the ITO electrode. A ±10-mV vs. Ag/AgCl 9-MHz triangular wave potential was applied to the electrode for 60 min in PBS(−) at RT

After negative potential application in non-nutritive solutions for 24 h, almost none of the soil microorganisms attached to the electrode surface could be detached by scraping several times with a rubber cell scraper. Therefore, we examined the electrical detachment and retrieval methods. In our previous study, we succeeded in detaching animal cells from an ITO electrode surface treated with extracellular matrix proteins after application of ±10 mV vs. Ag/AgCl 9-MHz triangular wave potential in PBS(−) for 30–60 min (Koyama 2011). Triangular wave potential-induced animal cell detachment is almost completely non-cytotoxic, and no statistically significant differences in HeLa cell growth were observed after they were subjected to trypsinization and high-frequency wave potential application (Koyama 2011). Therefore, we investigated whether high-frequency triangular wave potential application also induced the detachment of microorganisms from the ITO electrode surface. Figure 6a shows the electrical detachment of soil microorganisms, *E*. *coli*, and *B*. *subtilis* from the patterned ITO electrode. After the negative potential induced microorganism attachment and the electrode surface was washed with PBS(−), ±10-mV vs. Ag/AgCl 9-MHz triangular wave potential was applied to the microorganisms on the ITO electrode in fresh PBS(−) for 60 min at RT. High-frequency triangular wave potential induced detachment of soil microorganisms, *E*. *coli*, and *B*. *subtilis* from the ITO electrode after 60 min of application (Fig. 6a). Figure 6b shows the time course of soil microorganism detachment from the reticulated ITO electrode region. Only PBS(−) treatment for 60 min did not detach the microorganisms from the electrode surface (Fig. 6b). A ±10-mV vs. Ag/AgCl 9-MHz triangular wave potential detached 66 % of the soil microorganisms after 30 min of application, and almost all of the microorganisms were detached after 60 min of application (Fig. 6b). Under the experimental conditions shown in Fig. 6, we collected 3.0 × 10^9^ cells/g (1.5 × 10^5^ cells/50 μg) of living microorganisms from the total 4.2 × 10^9^ cells/g in the garden soil sample. Table 1 shows the viability of the microorganisms after high-frequency triangular wave potential application. The application of ±10, ±6, and ±4 mV vs. Ag/AgCl 9-MHz triangular wave potential detached more than 98 % of the microorganisms on the electrode surface after 60 min in PBS(−) at RT (Table 1). To examine the cytotoxicity of electrical detachment using the high-frequency triangular wave potential, we measured the survival rates of the residual microorganisms on the electrode surface after 60 min of application. High and low survival rates were 91 % (517 of 566 cells) and 17 % (120 of 726 cells) of soil microorganisms after ±10 and ±8 mV 9-MHz triangular wave potential application, respectively (Table 1). After ±8 mV triangular wave potential application for 60 min, a large portion of the remaining microorganisms on the electrode surface was comprised of dead cells, and the detachment rate was lower than under the other experimental conditions (Table 1). In the cultured microorganisms, 83 % (283 of 342 cells) of the *E*. *coli* and 47 % (243 of 522 cells) of the *B*. *subtilis* cells remained alive after 60 min of application (Table 1). We also examined the viable bacteria collection rates of *E*. *coli* and *B*. *subtilis* by colony counting in the supernatants in the three-electrode chamber system (Table 1). We collected the supernatants of both after 24 h of application of the −0.4 V vs. Ag/AgCl potential and after 1 h of application of the high-frequency triangular wave potential. The viable bacteria collection rate was calculated from the colony number after high-frequency triangular wave potential application divided by the total colony number. The viable bacteria collection rates indicated similar survival rates (Table 1). We successfully recovered 88 % (238 of 272 colony-forming units (CFU)) of *E*. *coli* and 48 % (157 of 328 CFU) of *B*. *subtilis* from the cell suspensions using the electrical attachment and detachment techniques (Table 1).

**Table 1:** Viability analyses of electrically detached microorganisms

Sample^a^	Triangular wave potential, 1 h at RT	Detachment rate (%)^b^	Survival rate (%)^c^	Viable bacteria collection rate (%)^d^
Garden soil	±10 mV, 9 MHz	99.8	91	N. D.
Garden soil	±8 mV, 9 MHz	65.5	17	N. D.
Garden soil	±6 mV, 9 MHz	99.8	58	N. D.
Garden soil	±4 mV, 9 MHz	98	38	N. D.
*E*. *coli*	±10 mV, 9 MHz	99.6	83	88
*B*. *subtilis*	±10 mV, 9 MHz	99.7	47	48

*N*. *D*. not determined

^a^Each microorganism in PBS(−) selectively attached to the reticulated ITO electrode region to which −0.4 V vs. Ag/AgCl potential was applied for 24 h at RT. Then, ±10 mV vs. Ag/AgCl 9-MHz triangular wave potential was applied for 1 h at RT

^b^Cell numbers were counted in randomly selected 50 × 50-μm^2^ regions of the reticulated ITO electrode region before and after triangular wave potential application

^c^More than 300 residual microorganisms on the electrodes were distinguished using a live/dead backlight bacterial viability kit after electrical detachment. Survival rates were determined by the sum of three independent experiments

^d^The viable bacteria collection rate was calculated from colony numbers after triangular wave potential application divided by the total colony number. Collection rates were determined by the sum of three independent experiments

### Electrical Collection of Microorganisms from Deep-Sea Sediment

**Fig. 7:**
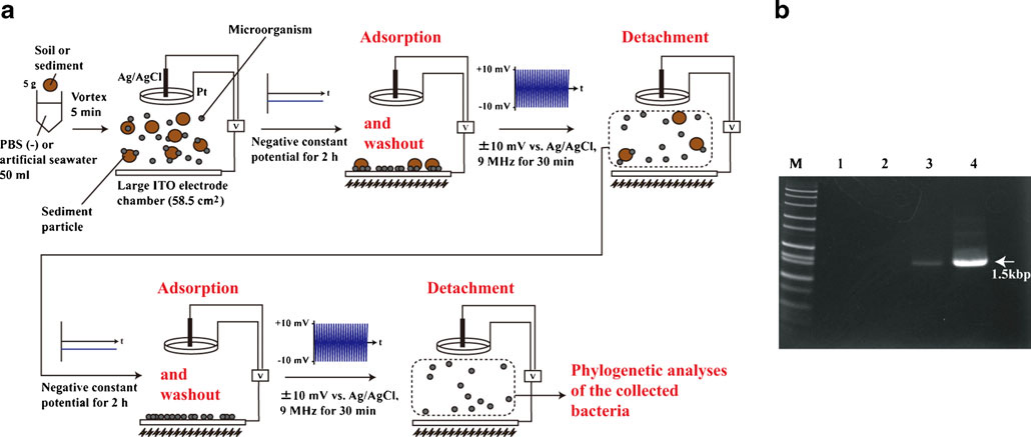
Electrical retrieval method for soil or sediment microorganisms. **a** Schematic illustration of the electrical retrieval method for soil or sediment microorganisms. **b** PCR products from the garden soil samples before purification of phylogenetic analyses. *M* DNA marker, *1* negative control, *2* direct soil DNA extraction, *3* first electrically retrieved microorganisms derived DNA, *4* second electrically retrieved microorganisms derived DNA

**Fig. 8:**
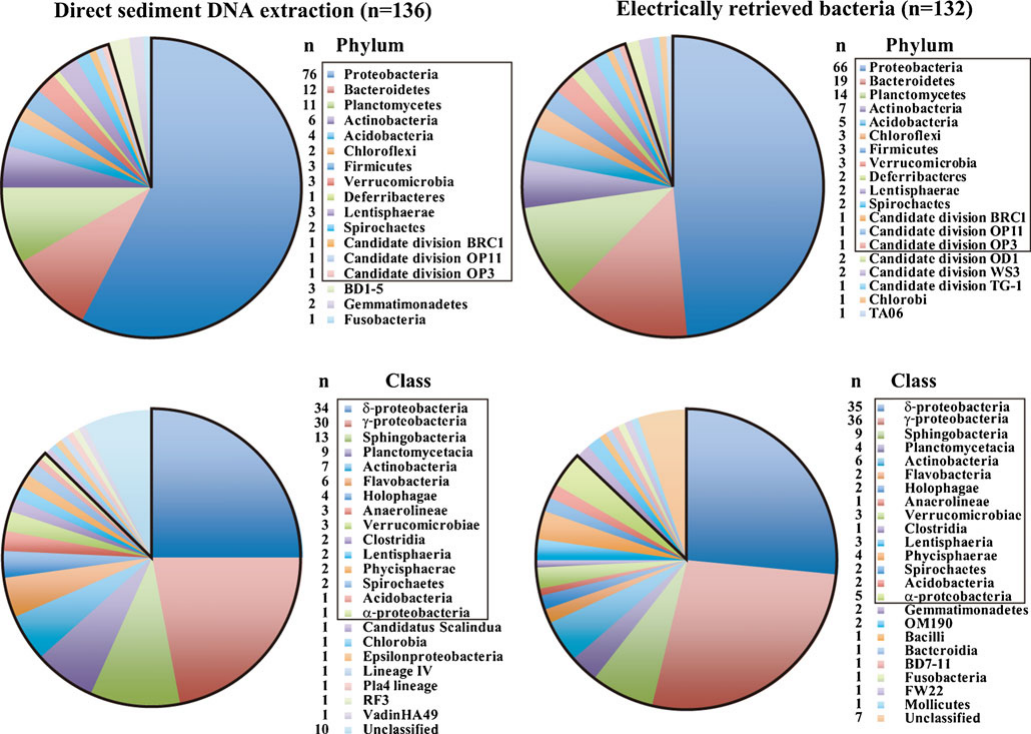
Phylogenetic affiliation of strains isolated from deep-sea sediment. The outlined regions indicate common phylotypes between direct sediment DNA extraction and electrically retrieved bacteria

To determine which types of microorganism would attach to the electrode surface with a negative potential applied, we used a deep-sea sediment sample collected at a depth of 1,176 m in the seep area of Sagami Bay, Japan (Koyama and Aizawa 2000; Koyama et al. 2005). The microbial flora composition of the microorganisms electrically retrieved from the sediment samples was compared with that of the original sample by phylotype analysis of PCR-amplified 16S rRNA genes (Figs. 7 and 8). In the garden soil sample, no PCR amplicon was obtained from the DNA directly extracted from the soil sample (Fig. 7). The PCR may be inhibited by humic substances in the soil (Roh et al. 2006), and therefore the microbial flora in the electrically retrieved garden soil microorganisms and DNA directly extracted from the soil sample could not be compared (Fig. 7). Figure 7a shows the method used for electrical retrieval of microorganisms from the deep-sea sediment samples for phylogenetic analyses. To determine the microbial composition from the sediment sample, the electrical retrieval of microorganisms was performed twice using the large electrode chamber devices (Fig. 7a). Moreover, both the constant and high-frequency triangular potential application times were shortened (Fig. 7a). After the procedures shown in Fig. 7a, we obtained 1.3 × 10^8^ microorganisms from the 5-g deep-sea sediment sample. Figure 8 compares the microbial community structure of the deep-sea sediment after electrical retrieval method and direct sediment DNA extraction. Deep-sea microorganisms belonging to 19 phyla and 23 classes were obtained using the electrical retrieval method (Fig. 8). The majority of the retrieved clones obtained with the electrical retrieval method were affiliated with *Proteobacteria* (Fig. 8). The remaining phyla were affiliated with *Bacteroidetes*, *Planctomycetes*, *Actinobacteria*, *Acidobacteria*, *Chloroflexi*, *Firmicutes*, *Verrucomicrobia*, *Deferribacteres*, *Lentisphaerae*, *Spirochaetes*, *Candidate division* BRC1, OP11, OP3, OD1, WS3, TG-1, *Chlorobi*, and TA06 groups (Fig. 8). At the phylum level 95 % and at the class level 87 % of the phylotypes among electrically retrieved bacteria were common to the gene clones from the direct sediment DNA extraction (Fig. 8). The results in Fig. 8 indicate that the electrical retrieval method collected a broad range of living microorganisms, which may reflect the organization of the microbial community in the sediment sample.

## Discussion

In this study, we demonstrated that an extensive range of living bacteria attached to the electrode surface with a negative applied potential in non-nutritive media. This result was supported by the *E*. *coli* attachment results (Fig. 2); the AFM and SEM images of *E*. *coli* (Fig. 3); the *B*. *subtilis*, *B*. *halodurans*, *K*. *rosea*, *S*. *violacea*, *S*. *oneidensis*, and soil microorganism attachment results (Figs. 4 and 5); and the determination of the deep-sea microbial community structure (Fig. 8). Nineteen phyla and 23 classes of microorganisms in deep-sea sediment samples were collected using the electrical attachment and detachment methods (Fig. 8). The electrical retrieval method collected almost all of the bacterial phylotypes detected using the direct DNA extraction method (Fig. 8). Furthermore, the electrical modulation of the spatial configuration of *E*. *coli* cells was also successful using ITO microelectrodes (Fig. 3). These techniques hold potential for novel microbial metagenomic analyses.

The results in Figs. 2, 3, 4, and 8 suggest that the ITO electrode region with a negative applied potential acts as an energy source for a broad range of microorganisms. It appears that microorganisms such as *E*. *coli*, *B*. *subtilis*, *B*. *halodurans*, *K*. *rosea*, *S*. *violacea*, and *S*. *oneidensis* produce adherent short fibrous materials in response to contact with the electrode surface with a negative applied potential (Figs. 2, 3, and 4). We observed a weak electrical current of −0.19 to −0.88 μA/cm^2^ when a negative constant potential between −0.3- and −0.4 V vs. Ag/AgCl was applied to cultured microorganisms and soil and sediment samples. The pili of *Geobacter sulfurreducens* may serve as biological nanowires, transferring electrons from the cell surface to the surface of Fe(III) oxides (Reguera et al. 2005). *S*. *oneidensis* nanowires were found to be electrically conductive along micrometer-length scales, with a measured resistivity on the order of 1 Ω cm (El-Naggar et al. 2010). Electron donors are often called energy sources because energy is released when they are oxidized (Madigan and Martinko 2006). Therefore, an electrode with a negative applied potential per se may act as an electron donor that is available for a variety of microorganisms. If the electrode acts as an electron donor in PBS(−), oxygen would be an electron acceptor (Fig. 2). Under anaerobic and PBS(−) conditions, the maximum attached *E*. *coli* cell density was increased (Fig. 2b, c). To obtain the anaerobic conditions, we used the anaerobic cultivation system that maintained 5 % carbon dioxide and less than 1 % oxygen concentrations after 60 min of cultivation. Although the *E*. *coli* cell density was enhanced in PBS(−), no statistically significant differences between the −0.4 V vs. Ag/AgCl application and the open circuit were observed in 280 mM glucose under both aerobic and anaerobic conditions (Fig. 2b, c). Therefore, the microorganisms attached on the negative potential applied electrode surface might synthesize a part of microbial components from carbon dioxide. The −0.3 and −0.4 V vs. Ag/AgCl negative potentials correspond to −0.1 and −0.2 V of reduction potentials (*E*_0_′), respectively. If the microorganisms on the negative potential applied electrode induce carbon dioxide fixation, energy supply of ATP molecules would be needed for the fixation reactions because of the low reduction potentials of the electrode (Madigan and Martinko 2006). Further research will determine the mechanisms on how the negative potential induced a wide range of the microorganism attachments. The electrical retrieval method could be used not only for microorganism extraction from soil and sediment samples but also for symbiotic bacteria extraction from biological samples. The electrical modulation method would also expand applications of the microbial battery and sensor in diverse ways.

In our previous study using animal cells, we found that ±10-mV vs. Ag/AgCl 9-MHz triangular wave potential induced detachment of both animal cells and extracellular matrix proteins (Koyama 2011). The mechanism of detachment involved both oscillation of the negative zeta potential-charged animal cells and the insertion of water molecules between the electrode surface and extracellular matrix proteins resulting from increments in hydrophilicity on the electrode surface (Koyama 2011). Because the triangular wave potential oscillated and detached the negative zeta potential-charged cells, potential application of optimal wave shape, optimal resonance frequency, and optimal amplitude in each phylotype of microorganism would shorten the time to detachment and retrieval of living microorganisms from the electrode (Fig. 6; Table 1).
